# Alpha1-Adrenergic Receptor Mediated Long-Term Depression at CA3-CA1 Synapses Can Be Induced *via* Accumulation of Endogenous Norepinephrine and Is Preserved Following Noradrenergic Denervation

**DOI:** 10.3389/fnsyn.2019.00027

**Published:** 2019-10-09

**Authors:** Katie Dyer-Reaves, Anthoni M. Goodman, Amy R. Nelson, Lori L. McMahon

**Affiliations:** ^1^Department of Cell, Developmental, and Integrative Biology (CDIB), School of Medicine, University of Alabama at Birmingham, Birmingham, AL, United States; ^2^Department of Psychology, University of Alabama at Birmingham, Birmingham, AL, United States

**Keywords:** hippocampus, norepinephrine, LTD, α_1_-AR, locus coeruleus

## Abstract

Locus coeruleus (LC) provides the sole source of noradrenergic (NA) innervation to hippocampus, and it undergoes significant degeneration early in Alzheimer’s disease (AD). Norepinephrine (NE) modulates synaptic transmission and plasticity at hippocampal synapses which likely contributes to hippocampus-dependent learning and memory. We previously reported that pharmacological activation of α_1_ adrenergic receptors (α_1_ARs) induces long-term depression (LTD) at CA3-CA1 synapses. Here, we investigated whether accumulation of endogenous NE *via* pharmacological blockade of norepinephrine transporters (NETs) and the NE degradative enzyme, monoamine oxidase (MAO), can induce α_1_AR LTD, as these inhibitors are used clinically. Further, we sought to determine how degeneration of hippocampal NA innervation, as occurs in AD, impacts α_1_AR function and α_1_AR LTD. Bath application of NET and MAO inhibitors in slices from control rats reliably induced α_1_AR LTD when β adrenergic receptors were inhibited. To induce degeneration of LC-NA innervation, rats were treated with the specific NA neurotoxin DSP-4 and recordings performed 1–3 weeks later when NA axon degeneration had stabilized. Even with 85% loss of hippocampal NA innervation, α_1_AR LTD was successfully induced using either the α_1_AR agonist phenylephrine or the combined NET and MAO inhibitors, and importantly, the LTD magnitude was not different from saline-treated control. These data suggest that despite significant decreases in NA input to hippocampus, the mechanisms necessary for the induction of α_1_AR LTD remain functional. Furthermore, we posit that α_1_AR activation could be a viable therapeutic target for pharmacological intervention in AD and other diseases involving malfunctions of NA neurotransmission.

## Introduction

Noradrenergic (NA) input from the locus coeruleus (LC) to hippocampus is critical for hippocampus-dependent learning and memory (Koob et al., 1978; Harro et al., 1999; Lemon et al., 2009; Gibbs et al., 2010), and its degeneration in Alzheimer’s disease (AD) has been well documented (Forno, 1966; Yamada and Mehraein, 1977; Zarow et al., 2003). Specifically, loss of LC cell bodies positively correlates with the severity of dementia and duration of the disease in AD patients. In fact, the LC is the first target of pretangle tau pathology, with measurable cell loss occurring in the prodromal phase of AD (Grudzien et al., 2007; Braak et al., 2011; Jucker and Walker, 2011; Arendt et al., 2015; Chalermpalanupap et al., 2017) that may mediate the emergence of cognitive changes (Braak et al., 2011; Arendt et al., 2015; Chalermpalanupap et al., 2017; Kelly et al., 2017; Theofilas et al., 2017). Despite this body of evidence, the effect of early LC degeneration on hippocampal synaptic function is poorly understood.

The LC is the sole provider of NA innervation in hippocampus (Aston-Jones, 2004), and norepinephrine (NE) modulates synaptic efficiency critical for learning and memory (Harley, 1991, 2007; Scheiderer et al., 2004; Kemp and Manahan-Vaughan, 2008; Hagena et al., 2016). Selective electrolytic lesioning using the neurotoxin N-(2-chloroethyl)-N-ethyl-2-bromobenzylamine (DSP-4) or silencing of LC neurons using optogenetics in murine models leads to deficits in learning, memory, and cognitive flexibility (Koob et al., 1978; Harro et al., 1999; Janitzky et al., 2015). NE, *via* activation of adrenergic receptors (ARs), modifies the strength of synaptic transmission at glutamatergic synapses and the ability of these synapses to undergo long-term plasticity (Hopkins and Johnston, 1984; Harley, 1991; Bröcher et al., 1992; Harley and Sara, 1992; Bramham et al., 1997; Erickson et al., 1997; Katsuki et al., 1997; Thomas and Palmiter, 1997a,b,c; Izumi and Zorumski, 1999). Accordingly, blockade of β adrenergic receptors (ARs) reduces NMDAR-dependent LTP in hippocampal slices (Harley, 1991), while NE application facilitates the induction of LTP in dentate and spatial memory formation through activation of β-ARs (Hopkins and Johnston, 1984; Bröcher et al., 1992; Katsuki et al., 1997; Izumi and Zorumski, 1999; Kemp and Manahan-Vaughan, 2008; André et al., 2015). Along with β-ARs, α-ARs are necessary for spatial memory learning tasks, as α_1_AR agonists enhance and antagonists block the formation of memory (Pussinen et al., 1997; Puumala et al., 1998). Activation of α_1_- and β-ARs by NE can also facilitate tetanus-induced LTP at mossy-fibers synapses in area CA3 (Hopkins and Johnston, 1984; Huang et al., 1996). Transgenic mice harboring constitutively active α_1_ adrenergic receptors (α_1_ARs) have enhanced learning and memory, while α_1_AR knock-out mice showed deficits compared to WT (Doze et al., 2011; Collette et al., 2014). We previously reported that NE, or the selective α_1_AR agonist methoxamine, induces a form of long-term depression (LTD) at CA3-CA1 synapses that is activity and NMDA receptor-dependent, and also requires activation of Src kinase and an increase in extracellular signal-regulated protein kinase (ERK) activation (Scheiderer et al., 2004, 2008). During expression of α_1_AR LTD, there is no change in the paired-pulse ratio compared to baseline, consistent with a post-synaptic locus of expression (Scheiderer et al., 2004). Furthermore, α_1_AR LTD shares the same mechanism as a form of LTD induced by activation of M1 muscarinic receptors (mAChRs), which are also coupled to Gq signaling. When weak activation of α_1_ARs is paired with weak activation of M1 mAChRs, LTD can be successfully induced, demonstrating the shared signaling pathways (Scheiderer et al., 2008).

Given that activation of α_1_ARs using exogenous agonists induces LTD at hippocampal CA3-CA1 synapses (Scheiderer et al., 2004, 2008), we wanted to determine if increasing endogenous extracellular NE accumulation *via* pharmacological inhibition of the norepinephrine transporter (NET) and the degradative enzyme monoamine oxidase (MAO) similarly induces α_1_AR LTD. This is important since these inhibitors are widely used as therapeutic treatments in disorders such as ADHD and depression, where imbalances in catecholamine neurotransmission, specifically NE, are known to occur (Zametkin and Rapoport, 1987; Castellanos et al., 1996; Vanicek et al., 2014; Israel, 2015). Furthermore, because LC degeneration, and loss of hippocampal NA innervation, is clinically relevant to normal aging, AD, and Parkinson’s disease (PD; Mann, 1983; Mann et al., 1983; Marien et al., 2004; Szot, 2012), we set out to investigate the impact of NA degeneration on the ability of pharmacological activation of α_1_ARs to induce LTD at hippocampal CA3-CA1 synapses.

## Materials and Methods

### Animal Care

All experiments were conducted with an approved protocol from the University of Alabama at Birmingham Institutional Animal Care and Use Committee in compliance with the National Institutes of Health guidelines. All efforts were made to minimize animal suffering and to reduce the number of animals used. Six-week-old male Sprague–Dawley rats (Charles River) were used in all experiments. Animals were housed two per cage and were kept on a 12-h light/dark cycle with *ad libitum* food and water.

### LC Lesion

Hippocampal NA denervation was performed using the NA axon specific neurotoxin DSP-4 (Tocris, Ellisville, MO, USA), known to cause terminal retrograde degeneration by targeting the NE uptake system (Jaim-Etcheverry and Zieher, 1980; Ross and Stenfors, 2015). DSP-4, delivered intraperitoneally (IP), readily crosses the blood-brain-barrier and targets NA axons of the central nervous system while peripheral NA systems are unaffected (Jaim-Etcheverry and Zieher, 1980; Fritschy and Grzanna, 1989; Ross and Stenfors, 2015). NE uptake in these axons is rapidly blocked with maximum effect achieved by 4–6 h and within 4–5 days DβH immunoreactivity declines (Ross, 1976; Fritschy et al., 1990). NA sprouting occurs in a region-specific manner over approximately 5 weeks (Booze et al., 1988) along with an observed 57% decline in LC cell bodies 1 year after DSP-4 treatment (Fritschy and Grzanna, 1992). Rats were lightly anesthetized with isofluorane and injected IP with DSP-4 (50 mg/kg) in saline or saline alone at 48-h intervals for a total of three injections.

### Drugs and Solutions

All drugs (Sigma, St. Louis, MO, USA) were prepared as stock solutions and diluted to the appropriate working concentration at the time of electrophysiology. Phenylephrine (Phe, α_1_AR agonist; in deionized water), propranolol (β-AR antagonist; in DMSO) and prazosin (α_1_AR antagonist; in DMSO) were prepared fresh daily and atomoxetine (Atmx, NET inhibitor; in deionized water) and clorgiline (Clor, MAO inhibitor; in deionized water) were frozen in 300 μL aliquots until used for recordings.

### Slice Preparation and Electrophysiological Recordings

α_1_AR LTD was induced using the α_1_AR agonist phenylephrine (Phe, 100 μM) that was bath applied for 10 or 15 min following a 20 min stable baseline of recorded field excitatory post-synaptic potentials (fEPSPs) as done previously (Scheiderer et al., 2004, 2008). Experiments to test whether extracellular accumulation of endogenous NE could induce LTD, as well as to test the functionality of the NA fibers remaining following DSP-4-mediated lesion, slices prepared from rats 7–8 days post-DSP-4 treatment were exposed to bath application of the NET inhibitor Atmx (500 nM) plus the MAO inhibitor Clor (10 μM) for 10 or 20 min following stable baseline transmission. Previous reports have documented the ability of NET inhibition to block reuptake of NE and induce increases in extracellular NE (Youdim and Riederer, 1993). Additionally, selective inhibition of MAO, the enzyme responsible for NE degradation, has also been shown to cause accumulation of NE extrasynaptically (Youdim and Riederer, 1993). Because NE has similar affinity for α- and β-ARs, it was necessary to pharmacologically inhibit β-AR activation with propranolol (10 μm) to reveal LTD following accumulation of extracellular NE. To ensure the α_1_AR specificity of the LTD induced by endogenous NE in these recordings, interleaved experiments were conducted in the presence of 10 μM prazosin (in addition to the cocktail of Clor, Atmx and propranolol, which is referred to as CAP).

### Immunohistochemistry

Following electrophysiological recordings, 400 μm-thick hippocampal slices were stored in 4% paraformaldehyde at 4°C until the time of staining. Twenty-four hours prior to staining, slices were rinsed in phosphate buffered saline (PBS) and then transferred to a 30% sucrose/PBS solution. Tissue was resectioned to 50 μm using a freezing microtome. Sections were washed 3 × 10 min in PBS at room temperature and incubated in blocking buffer [10% normal donkey serum (NDS) in 0.3% PBS Triton/PBS] for 90 min. Primary antibodies [rabbit anti-tyrosine hydroxylase (TH, 1:200) and mouse anti-dopamine β-hydroxylase (DβH, 1:300); Chemicon, Temecula, CA, USA] were diluted in blocking buffer, and applied to free-floating sections and incubated overnight at 4°C. Slices were washed 3 × 10 min with PBS and were labeled with fluorescence-activated secondary antibodies [donkey anti-rabbit Alexa 594 (1:200) and donkey anti-mouse Alexa 488 (1:200); Invitrogen, Eugene, OR, USA] diluted in blocking buffer and incubated for 1–2 h at room temperature. Slices were washed 3 × 30 min and incubated with Hoescht nuclear stain (1 μl stock/10 ml PBS) for 15 min at room temperature. Slices were mounted on slides using Permafluor (Immunon, Waltman, MA, USA) and viewed on a Leica (Exton, PA, USA) DM IRBE laser scanning confocal microscope. The CA1 s. radiatum within the field of view from one slice per animal was analyzed using ImageJ software. Sequential scans of blue, green, and red channels were obtained and ~20 μm stacks of images were collected in a z-axis of 1.0–1.5 μm step size, averaging two scans per image. Maximum projections were generated and used for NE fiber quantification. DβH-positive fibers were measured and counted, following the criterion that only fibers with four or more consecutive boutons be considered as a fragment of axon.

### Data Analysis

Electrophysiology data were analyzed using custom-written Labview data acquisition and analysis software (Scheiderer et al., 2004, 2008) after being filtered at 3 kHz and digitized at 10 kHz. The fEPSP slope was measured and evaluated as a series of five averaged raw data points plotted vs. time. The LTD magnitude was calculated by comparing the average fEPSP slope recorded during the last 10 min of baseline transmission to the slope at 20 min post-drug washout. When more than one slice was recorded per animal for a given experiment (e.g., Phe or Amtx ± Clor), the data were averaged together to represent the finding for that animal. Therefore, the reported *n* refers to animal number. Paired student’s *t-*tests were used for statistical analysis within groups. Unpaired student’s *t-*tests or one-way analysis of variances (ANOVAs) were used to evaluate statistical significance between groups. The significance level was set at *p* < 0.05 and the data are presented as the mean ± SEM.

## Results

### α_1_AR Activation Induces LTD at CA3-CA1 Synapses in Rat Hippocampus

**Figure 1:**
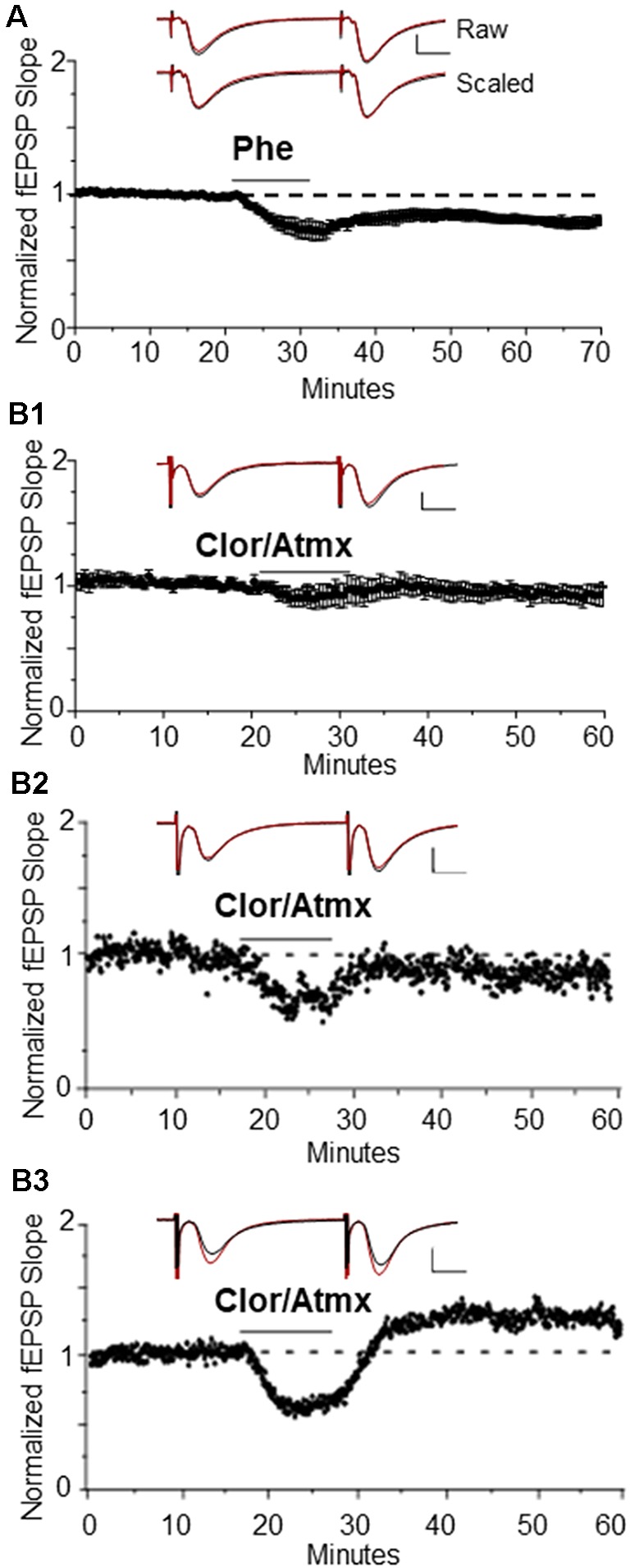
Long-term depression (LTD) is induced by the α_1_AR agonist, phenylephrine, or endogenous NE when β-ARs are pharmacologically blocked. **(A)** α_1_AR LTD is induced by the selective α_1_AR agonist Phe. One-hundred micro molar is able to induce α_1_AR LTD in control animals [84 ± 4% of baselined field excitatory postsynaptic potential (fEPSP) slope, *n*=6]. Top traces are averaged raw fEPSPs before (black) and after (red) application of phenylephrine. Bottom traces are averaged, scaled fEPSPs (red) during LTD expression overlaid onto baseline traces (black) showing no change in the pair-pulse facilitation ratio (scale bar: 0.5 mV, 10 ms). **(B1)** Averaged experiments in Clor/Atmx results in no significant change in baseline transmission (94 ± 5% of baseline fEPSP slope, *n* = 6). **(B2)** Representative example of LTD induced *via* norepinephrine transporter (NET) and monoamine oxidase (MAO) inhibition by Atmx (500 nM) and Clor (10 μM, respectively). **(B3)** Single example of LTP induced *via* endogenous NE accumulation in the presence of Clor/Atmx.

Our laboratory previously reported that bath application of NE or the selective α_1_AR agonist methoxamine is sufficient to induce a NMDAR-dependent LTD of extracellular fEPSPs at CA3-CA1 glutamatergic synapses in hippocampal slices (Scheiderer et al., 2004, 2008). Here, we show that application of another α_1_AR agonist, Phe (100 μM; phenylephrine) also reliably induces α_1_AR LTD [Figure 1A, 84 ± 4% of baseline fEPSP slope (*n* = 6); *p* < 0.01] similar to our previous report (Scheiderer et al., 2004, 2008). To test whether α_1_AR LTD can also be induced *via* extracellular accumulation of endogenous NE in hippocampus, the selective NET inhibitor Atmx (500 nM) was applied together with an inhibitor of NE degradative enzyme MAO, Clor (10 μM). Bath application of Atmx and Clor did not elicit a significant change in synaptic strength compared to baseline (Figure 1B1, 94 ± 5% of baselined fEPSP slope, *n* = 6, *p* = 0.14). However, when we evaluated individual experiments there were clear cases of Atmx and Clor induced depression (Figure 1B2) and potentiation (Figure 1B3) of the extracellular fEPSP that were masked when all of the experiments were averaged. This variable response can be attributed to coincident global activation of α_1_, α_2_, and β-ARs, as all of these receptors are located at pre- or post-synaptic locations at CA3-CA1 synapses.

**Figure 2:**
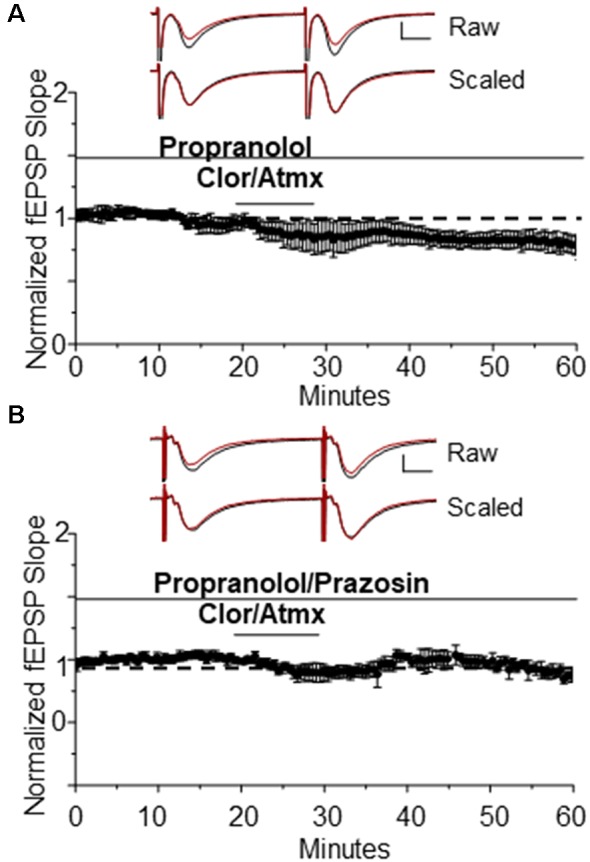
The β AR antagonist propranolol is able to unmask α_1_AR LTD when used in addition to Clor and Atmx. **(A)** In the presence of the β AR antagonist propranolol (1 μM) accumulation of endogenous NE induces α_1_AR LTD (83 ± 6%of baselined fEPSP slope, *n* = 4). Top traces are averaged raw fEPSPs before (black) and after (red) application of phenylephrine. Bottom traces are averaged, scaled fEPSPs (red) during LTD expression overlaid onto baseline traces (black) showing no change in the pair-pulse facilitation ratio (scale bar: 0.5 mV, 10 ms). **(B)** α_1_AR LTD is prevented by application of the α_1_AR antagonist prazosin (10 μM) in the presence of propranolol, Clor, and Atmx (*p* > 0.05).

Because activation of β-ARs causes potentiation at CA3-CA1 synapses (Hopkins and Johnston, 1984; Bröcher et al., 1992; Katsuki et al., 1997; Izumi and Zorumski, 1999), their activation by endogenous accumulation of NE could be masking possible α_1_AR LTD expression induced by Atmx and Clor application. To determine whether blockade of β-AR activation would unmask LTD, propranolol (10 μM) was applied for the duration of the recording period during the Atmx and Clor experiments (collectively named CAP), and this resulted in reliable, and significant LTD expression (Figure 2A, 83% ± 6% of baseline fEPSP slope, *n* = 4, *p* = 0.02). The LTD magnitude induced by the selective α_1_AR agonist Phe (Figure 1A) is not significantly different from that induced by the combination of NET, MAO, and β-AR inhibition (Figure 2A; *p* = 0.8). Importantly, when the raw traces during expression of LTD are scaled and overlaid with traces during baseline control conditions, it is clear that there is no difference in the paired-pulse facilitation ratio during LTD expressed induced either with phenylephrine or by CAP, similar to our previous reports (Scheiderer et al., 2004, 2008), suggesting a postsynaptic mechanism. To confirm that the LTD following accumulation of endogenous NE is also mediated by α_1_AR activation, interleaved experiments were performed with the α_1_AR antagonist prazosin (10 μM) resulting in a complete block of LTD (Figure 2B, CAP plus prazosin, 96.5 ± 4% of baseline fEPSP slope, *n* = 3, *p* = 0.4).

### DSP-4 Causes a Significant Decrease in NA Innervation in CA1 of Hippocampus

**Figure 3:**
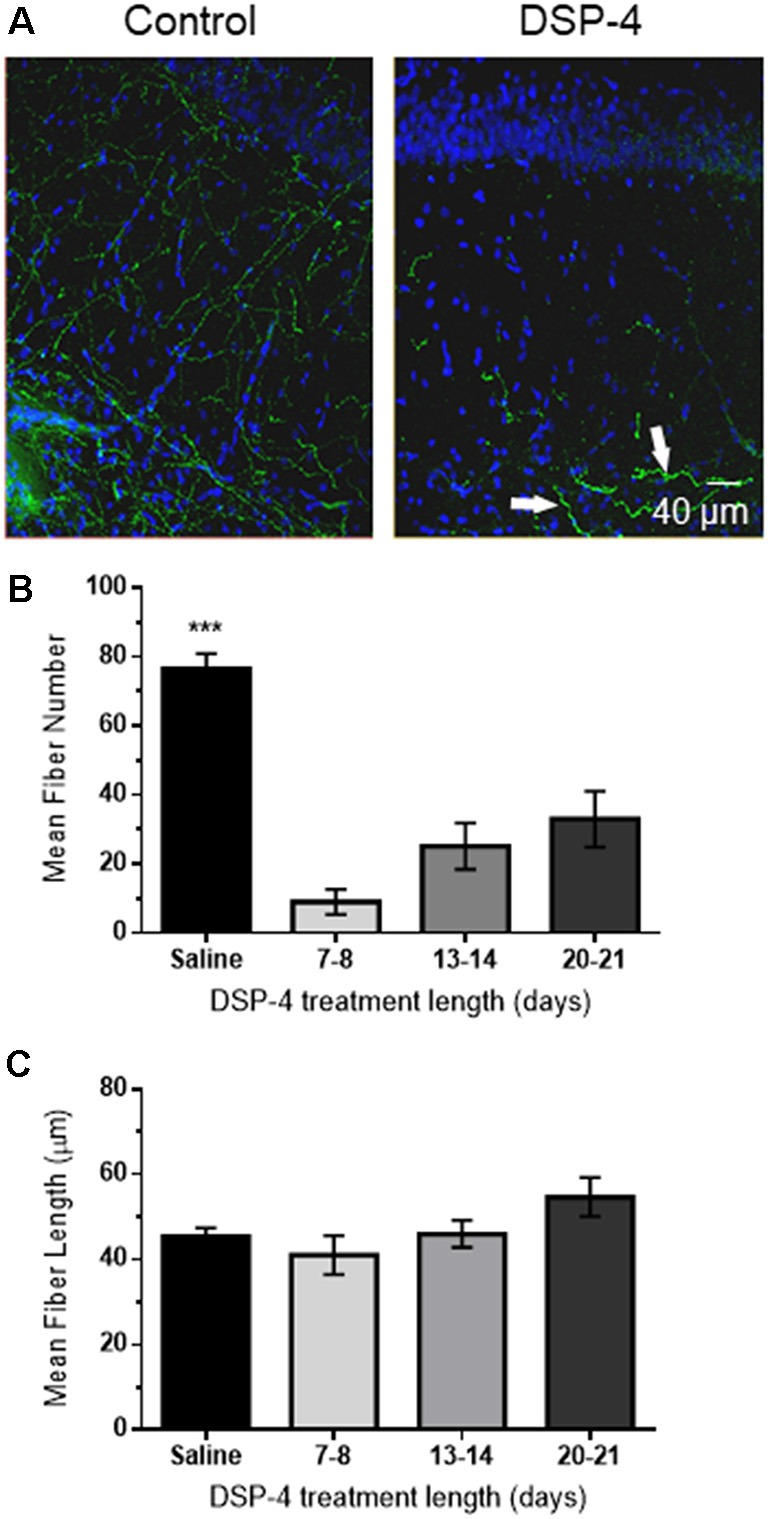
DSP-4 treatment causes significant NE degeneration in CA1 of hippocampus. **(A)** Anti-DβH (green) and DAPI (blue) hippocampal staining of NE fibers in area CA1 s. radiatum from a saline-treated (control) animal or following DSP-4 treatment (scale bar: 40 μm). Arrows denote thicker, knobby appearance of some remaining NE fibers. **(B)** Mean fiber number in DSP-4 treated animals in CA1 s. radiatum is significantly decreased compared to saline-treated measured 7–21 days post-treatment. **(C)** Mean NE fiber length is unchanged following DSP-4 treatment. **(C)** Total NE fiber number is significantly decreased by DSP-4 treatment by ~85% (saline, *n* = 7; DSP-4, *n* = 19).

To determine whether loss of NA input to hippocampus is sufficient to cause deficits in α_1_AR LTD, the NE specific neurotoxin DSP-4 (50 mg/kg, in saline), was administered intraperitoneally at 48-h intervals for a total of three injections (control animals received injections of saline only). DSP-4 targets the NE uptake system and induces alkylation of vital neuronal structures (Ross, 1976) causing degeneration of hippocampal NA innervation (Jonsson et al., 1981; Fritschy and Grzanna, 1989; Fritschy et al., 1990). This robust treatment protocol was used because a previous study reported that mice treated with one dose of DSP-4 had an increased probability of hippocampal NA axon sprouting compared to mice treated three times with the toxin (Puoliväli et al., 2000). NA innervation in s. radiatum of area CA1 following DSP-4 treatment was evaluated using anti-DβH immunohistochemical staining of NA fibers, which were then imaged *via* confocal microscopy. DSP-4 induced a significant decrease in NA fiber number (Figure 3B; *F*_(3,19)_ = 23.28, *p* < 0.001) but no change in individual fiber length in CA1 s. radiatum in animals sacrificed 7–21 days following the first injection (Figure 3C; *F*_(3,658)_ = 2.03, *p* = 0.108). This protocol led to a reduction of ~85% of DβH-positive immunostaining. Interestingly, the morphology of some of the remaining DβH-positive fibers in the DSP-4-treated animals have a thicker, knobby appearance compared to the thin, more delicate fibers found in the control group (Figure 3A, arrows).

### α_1_AR LTD Remains Intact Following NA-Fiber Degeneration

**Figure 4:**
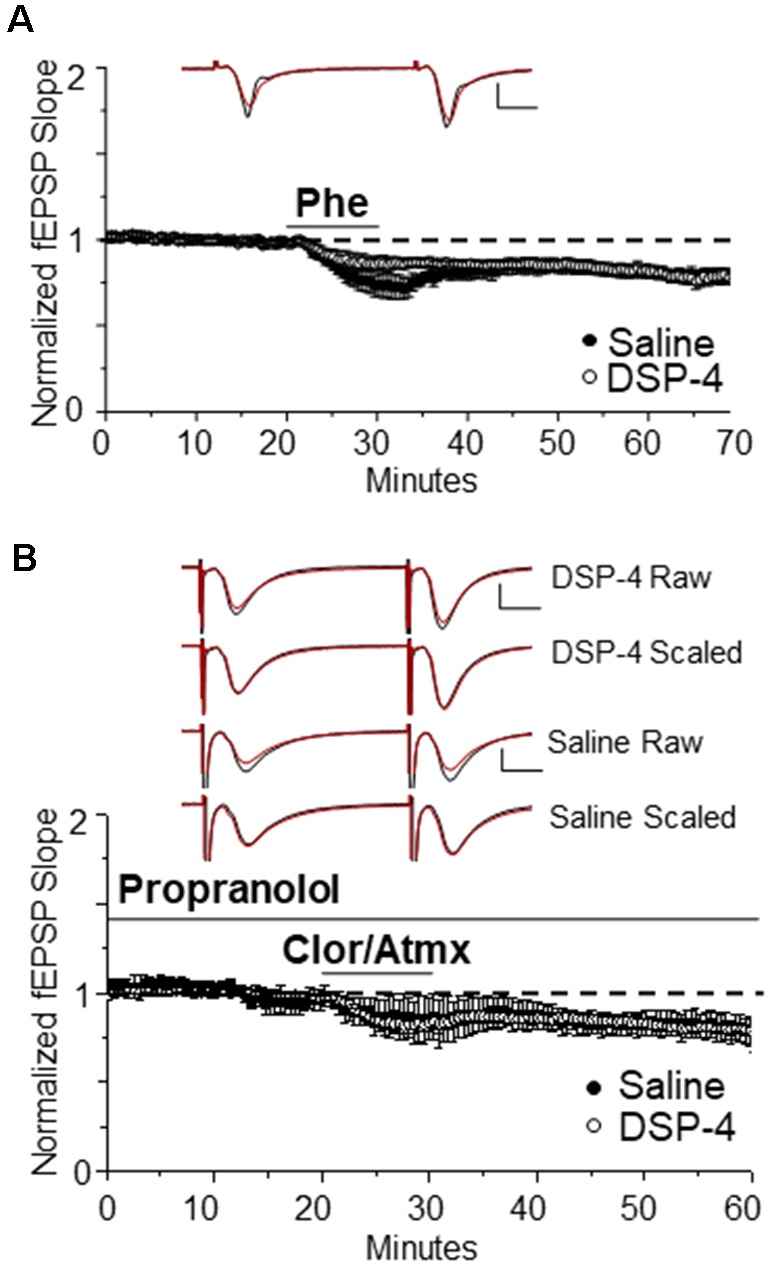
α_1_AR LTD remains intact following NE degeneration and is able to be induced by endogenous NE. **(A)** DSP-4 treatment does not prevent α_1_AR LTD (85% ± 3% of baselined fEPSP slope, *n* = 7). The magnitude of α_1_AR LTD induced by Phe is not significantly different between saline and DSP-4 treatment groups (*p* > 0.05; control data replotted from Figure 1A). Top traces are averaged raw fEPSPs before (black) and after (red) application of phenylephrine. Bottom traces are averaged, scaled fEPSPs (red) during LTD expression overlaid onto baseline traces (black) showing no change in the paired-pulse facilitation ratio (scale bar: 0.5 mV, 10 ms). **(B)** α_1_AR LTD is induced by endogenous NE accumulation in slices from DSP-4-treated rats (83 ± 3% of baseline fEPSP slope, *n* = 11). The magnitude of α_1_AR LTD induced by accumulation of endogenous NE is not significantly different between saline and DSP-4 treatment groups (*p* > 0.05; control data replotted from Figure 2A).

We next tested whether systemic treatment with DSP-4 and subsequent ~85% reduction of NA innervation would negatively impact the ability of direct activation of α_1_ARs by the selective α_1_AR agonist Phe to induce LTD. Surprisingly, we found that the magnitude of α_1_AR LTD was not significantly different between saline-treated and DSP-4 treated animals (Figure 4A, DSP-4: 85 ± 3%, *n* = 7, *p* < 0.001 vs. Figure 4B, saline vs. DSP-4, *p* = 0.85). Thus, despite an ~85% decrease in NA innervation in s. radiatum of area CA1, α_1_ARs remain coupled to downstream signaling cascades (Src kinase and pERK; Scheiderer et al., 2008) necessary for the induction of LTD. However, it is unclear whether α_1_AR LTD can be induced by endogenously released NE from the remaining 15% of NE fibers following DSP-4 treatment.

To determine whether the NA fibers surviving neurotoxic damage are able to functionally release NE and activate α_1_ARs effectively to induce LTD, NET, MAO, and β-ARs antagonists were bath applied. Again surprisingly, α_1_AR LTD was observed (Figure 4B, DSP-4: 83 ± 3%, *n* = 11, *p* < 0.001) and the magnitude was not different from saline-treated animals; (Figure 4B, saline vs. DSP-4, *p* = 0.97). Furthermore, the magnitude of LTD induced *via* activation of α_1_ARs by endogenously released NE was not significantly different from that *via* direct α_1_AR activation by Phe (Figures 4A,B, DSP-4, CAP vs. Phe *p* = 0.58).

**Figure 5:**
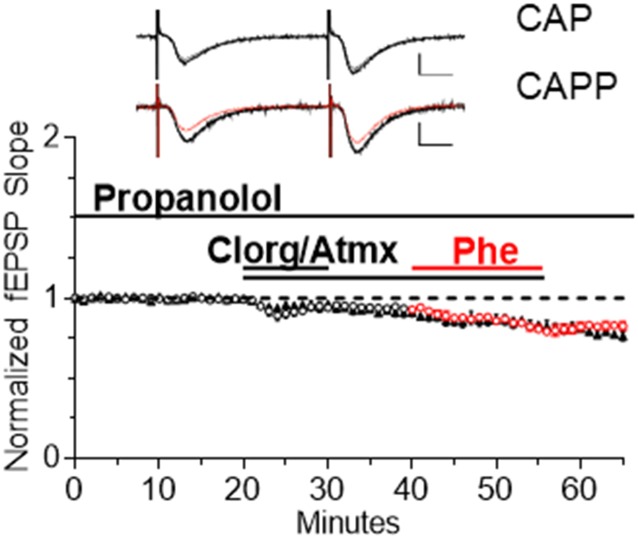
Endogenous NE accumulation is sufficient to cause maximal α_1_AR LTD. α_1_AR LTD is induced by endogenous NE accumulation in slices and subsequent application of Phe is unable to induce further LTD (CAP: *n* = 6; CAPP: *n* = 10, *t*_(6)_ = 2.08, *p* = 0.0827). Red data points denote the interleaved experiments in which the addition of Phe was included in the CAP mixture following 20 min of CAP application CAPP is CAP plus Phe.

To determine if α_1_AR LTD induced by endogenous NE release elicits maximal depression and occludes further depression induced by subsequent application of Phe, the drug mixture CAP was bath applied for 10 min to induce LTD. In interleaved slices, Phe (100 μM) was coapplied with CAP for 15 min beginning 20 min after CAP application to determine if additional LTD could be elicited (Figure 5). We found that no additional LTD could be induced by Phe in the presence of CAP, such that α_1_AR LTD induced by CAP that was not significantly different when Phe was added to the CAP mixture (denoted CAPP; Figure 5, CAP: *n* = 6; CAPP: *n* = 10, *t*_(6)_ = 2.08 *p* = 0.0827).

## Discussion

### α_1_AR LTD

Here, we have established that increasing extracellular accumulation of endogenously released NE can activate α_1_ARs and induce α_1_AR LTD at CA3-CA1 synapses in hippocampus. Furthermore, lesion of NA input to hippocampus from the LC did not prevent induction or expression of α_1_AR LTD, despite the loss of ~85% of hippocampal NA innervation. Surprisingly, the NA fibers that remained following DSP-4 induced lesion release enough NE that can accumulate following NET and MAO inhibition to activate postsynaptic α_1_ARs at CA3-CA1 synapses to induce LTD. NET and MAO inhibitors increase the accumulation of DA and 5-HT, especially in the hippocampus where NET serves as the primary reuptake mechanism for DA (Borgkvist et al., 2012). Although accumulation of DA and 5-HT are likely present, the LTD induced by NET and MAO inhibition was completely prevented by the α_1_AR antagonist prazosin, indicating that this LTD is dependent on activation of α_1_ARs. The blockade by prazosin also suggests that DA and 5HT must not be accumulating enough to induce LTD through D4 receptors (Navakkode et al., 2017) or 5HT4 receptors (Wawra et al., 2014). Therefore, these data suggest that in light of severe NE degeneration, α_1_ARs remain coupled to their signaling cascade and are able to be activated by specific α_1_AR agonists or *via* endogenously released NE from surviving NA fibers to induce LTD at CA3-CA1 synapses.

Previously, our lab has shown that M1 mAChRs LTD (mLTD) is lost following degeneration of hippocampal cholinergic innervation from the medial septum, but is rescued when hippocampal NA sympathetic sprouting occurs with an accompanying cholinergic reinnervation of hippocampus at 15% of control levels (Scheiderer et al., 2006). Because M1 mAChRs and α_1_ARs couple to the same G protein signaling pathway (Porter et al., 2002), and we have shown that their simultaneous weak pharmacological activation induces LTD (Scheiderer et al., 2008), we expected that loss of NA innervation to hippocampus would cause the same deficit in α_1_AR LTD as we have observed for M1 mAChR LTD following cholinergic degeneration in the absence of sprouting (Scheiderer et al., 2006). However, the successful expression of α_1_AR LTD at synapses in slices with 15% NA fibers remaining is consistent with the “rescued” mLTD by the approximate 15% cholinergic reinnervation stimulated by NA sympathetic sprouting after medial septal lesion. Thus, 15% of control cholinergic or NA innervation in hippocampus is enough to maintain the function of M1 mAChRs or α_1_ARs, respectively. Based on these findings, we speculate that α_1_ARs remain functional in AD and PD patients even with 67.9% and 83.2% cell loss (Marien et al., 2004). In fact, α_1_AR expression is increased in AD patients, but unfortunately, this has been associated with increased aggression in these patients (Szot et al., 2006; Sharp et al., 2007). Due to this, prazosin, an α_1_AR antagonist, has been used clinically in AD patients and has been shown to improve aggravation and aggression symptoms (Wang et al., 2010).

### DSP-4 Induced Lesion of Hippocampal NA Innervation

In humans, the severity of LC degeneration is positively correlated with intensity of AD symptoms and is detectable in the prodromal phase, prior to the onset of cognitive deficits (Grudzien et al., 2007; Braak et al., 2011; Arendt et al., 2015). While the onset of this degeneration is driven by tauopathy in humans (Hertz, 1989; Parvizi et al., 2001; Geula et al., 2008; Grinberg et al., 2009; Simic et al., 2009), we were able to recapitulate the effects of AD-driven LC degeneration with the neurotoxin, DSP-4 in healthy rats.

The DSP-4 treatment protocol employed reduced NA innervation by ~85%; additional injections or increases in DSP-4 concentration were not used due to observed increases in animal mortality (unpublished observations). It is important to note that the DSP-4 induced NA lesion is a variable model, as DSP-4 only provides a temporary decrease in NA innervation, whereas neurodegeneration is permanent. In fact, several studies have shown that DSP-4 induced NA degeneration is reversed several months following treatment (Fritschy and Grzanna, 1989, 1992; Fritschy et al., 1990). The LC-NA system is known to be capable of initiating these compensatory mechanisms in response to damage, which includes an increase in NE turnover (Jonsson et al., 1981) and release (Abercrombie and Zigmond, 1989) in surviving cell axons, as well as inducing α and β receptor supersensitivity (Dooley et al., 1983; Berridge and Dunn, 1990; Starke, 2001). Interestingly, AD patients have elevated NE levels even though there is LC cell loss (Szot et al., 2006), and α_2_-AR positive axonal sprouting has been identified in AD post-mortem human hippocampus (Szot et al., 2006). Thus, it can be postulated that excessive release, in addition to depletion, of NE could also lead to deficits in hippocampal-dependent learning and memory, and therefore, synaptic plasticity. Furthermore, the lesion protocol used here may cause increased activation of the NA compensatory mechanisms thought to be responsible for lesion reversal (Fritschy and Grzanna, 1992). In support of this idea, the DβH positive fiber morphology appears thicker and has a knobby appearance in the DSP-4 treated group compared to saline-treated control (Figure 3A, arrows). This morphology change could be an early indication of compensatory LC sprouting because as mentioned above, it has been shown that DSP-4 induced lesions are not permanent, and after a variable period of time hyperinnervation of NA fibers can occur (Booze et al., 1988; Fritschy and Grzanna, 1992; Kalinin et al., 2006). Altogether, we were unable to induce a complete loss of all NA hippocampal innervation using DSP-4 and were therefore unable to observe α_1_AR uncoupling and α_1_AR LTD loss that we predict would happen in the complete absence of endogenous NE, similar to our previous studies of M1 mAChR LTD following complete medial septal lesion (Scheiderer et al., 2006). It is interesting to note that uncoupling of α_1_AR function and loss of α_1_AR LTD occurs at glutamatergic synapses in mPFC in adult rats as a consequence of neonatal lesion of the ventral hippocampus (Bhardwaj et al., 2014).

The maintenance of α_1_AR function we observe following DSP-4 induced lesion could explain the inconsistent findings in learning and memory assays following DSP-4 treatment, with some studies reporting minimal effects (Prado de Carvalho and Zornetzer, 1981; Decker and McGaugh, 1991; Ohno et al., 1993, 1997), whereas others find deficits (Prado de Carvalho and Zornetzer, 1981; Decker and McGaugh, 1991). The reversibility of DSP-4 treatment, as well as variable treatment paradigms could lead to the confounding behavioral results following NA degeneration.

## Conclusion

Here, we show that pharmacological inhibition of NET and MAO leads to extracellular accumulation of NE which is capable of activating ARs that modulate synaptic efficacy at CA3-CA1 synapses. This finding is important, as NET and MAO inhibitors are used clinically in the treatment of several neurological and neuropsychiatric illness (Zametkin and Rapoport, 1987; Castellanos et al., 1996). Furthermore, the DSP-4-driven loss of ~85% of NA fibers in hippocampus recapitulates the loss of cortical projecting LC-cells in human AD (German et al., 1992; Grudzien et al., 2007; Braak et al., 2011; Arendt et al., 2015). The present findings suggest that α_1_ARs are preserved in CA3-CA1 glutamatergic synapses following extensive NA fiber denervation. This preservation of α_1_AR function may be due to compensatory NE levels released from remaining fibers or may suggest that these receptors are resilient to change even under conditions of chronically low stimulation. Thus, possible use of NET and MAO inhibitors in the treatment of AD will need much more investigation, as chronic elevation of NE may lead to receptor desensitization, and the role of each α and β AR subtype in modulating hippocampal function and learning and memory is complex.

## Data Availability Statement

The raw data supporting the conclusions of this manuscript will be made available by the authors, without undue reservation, to any qualified researcher.

## Ethics Statement

The animal study was reviewed and approved by University of Alabama at Birmingham Institutional Care and Use Committee.

## Author Contributions

KD-R and LM conceived and designed the study. KD-R and AG contributed to the execution of the experiments and performed the statistical analyses. KD-R wrote the first draft of the manuscript. AN, AG and LM wrote sections of the manuscript. All authors contributed to the manuscript revision, read and approved the submitted version.

## Conflict of Interest

The authors declare that the research was conducted in the absence of any commercial or financial relationships that could be construed as a potential conflict of interest.

## Abbreviations

AD: Alzheimer’s disease
AR: adrenergic receptor
Atmx: atomoxetine
Clor: clorgiline
DβH: dopamine β-hydroxylase
DSP-4: N-(2-chloroethyl)-N-ethyl-2-bromobenzylamine
fEPSP: field excitatory postsynaptic potential
LTD: long-term depression
MAO: monoamine oxidase
NA: noradrenergic
NE: norepinephrine
NET: norepinephrine transporter
Phe: phenylephrine
PD: Parkinson’s disease
s. radiatum: stratum radiatum
TH: tyrosine hydroxylase.
